# Genicular Artery Embolisation in Patients with Osteoarthritis of the Knee (GENESIS 2): Protocol for a Double-Blind Randomised Sham-Controlled Trial

**DOI:** 10.1007/s00270-023-03477-z

**Published:** 2023-06-19

**Authors:** Mark W. Little, Richard Harrison, Sarah MacGill, Archie Speirs, James H. Briggs, Edward Tayton, Nev L. C. Davies, Heike S. Hausen, Claire McCann, Lisa L. Levine, Ricky A. Sharma, Matthew Gibson

**Affiliations:** 1 University Department of Radiology, Royal Berkshire Hospital, London Road, Reading, RG1 5AN UK; 2 Centre for Integrative Neuroscience and Neurodynamics, University of Reading, Reading, UK; 3 Department of Orthopaedics, Royal Berkshire Hospital, Reading, UK; 4 Varian, a Siemens Healthineers Company, Palo Alto, USA

**Keywords:** Knee, Knee joint, Arthralgia, Osteoarthritis, Embozene™, Microspheres, Embolisation, Artery embolisation, Embolic agent, Arteries, Interventional radiology, Chronic pain

## Abstract

Knee osteoarthritis is a leading cause of chronic disability and economic burden. In many patients who are not surgical candidates, existing treatment options are insufficient. Clinical evidence for a new treatment approach, genicular artery embolisation (GAE), is currently limited to single arm cohort, or small population randomised studies. This trial will investigate the use of a permanent embolic agent for embolisation of abnormal genicular arterial vasculature to reduce pain in patients with mild to moderate knee osteoarthritis. Up to 110 participants, 45 years or older, with knee pain for ≥ 3 months resistant to conservative treatment will be randomised (1:1) to GAE or a sham procedure. The treatment group will receive embolisation using 100-micron Embozene™ microspheres (Varian, a Siemens Healthineers Company) (investigational use for this indication in the UK), and the sham group will receive 0.9% saline in an otherwise identical procedure. Patients will be followed for 24 months. At 6 months, sham participants will be offered crossover to GAE. The primary endpoint is change of 4 Knee Injury and OA Outcome Score subscales (KOOS_4_) at 6 months post-randomisation. The study will also evaluate quality of life, health economics, imaging findings, and psychosocial pain outcomes. The primary manuscript will be submitted for publication after all participants complete 6 months of follow-up. The trial is expected to run for 3.5 years.

* Trial Registration*: ClinicalTrials.gov, Identifier: NCT05423587.

**Supplementary Information:**

The online version contains supplementary material available at 10.1007/s00270-023-03477-z.

## Introduction

Knee osteoarthritis (OA) is a leading cause of chronic disability and economic burden [1]. Many patients with knee OA are resistant to conservative treatment options (analgesia, physiotherapy, and steroid injections) and may not yet be suitable for surgery. A growing body of evidence supports genicular artery embolisation (GAE) as a potential treatment option for this cohort of patients. GAE is a novel procedure in which abnormal vasculature arising from branches of the genicular arteries is embolised [2–9]. There are established feasibility and safety using permanent microspheres in patients with mild to moderate knee OA [6, 7]. Early results indicate that GAE may reduce both synovitis and OA symptoms [6]. Despite the growing body of evidence, generalisability is limited by small sample size, short follow-up, and lack of control groups to enable assessment of the placebo effect [10]. Furthermore, previous data have identified subsets of patients who do not report clinical benefit following GAE, despite an apparently technically successful procedure [6]. Emerging pain literature has explored whether specific neural and/or behavioural factors predispose to the central facilitation of pain and may therefore be able to predict treatment response following GAE [11].

The rationale of the current study is to improve data generalisability on the effectiveness of GAE, whilst testing the hypothesis that GAE is more effective than a sham procedure in treating mild to moderate knee OA.

## Materials and Methods

Genesis II is a double-blind randomised sham-controlled superiority trial that will test the hypothesis that GAE is more effective than a sham procedure. The study will also assess participant neuropsychological factors in an attempt to better understand how chronic pain affects treatment response to GAE. Contrast-enhanced MRI of the affected knee will be performed to assess imaging markers of success. Safety, cost-effectiveness, analgesia use, and patient reported outcome measures (PROMS) will all be investigated as part of this study. The study has full ethical approval (IRAS study number: 286849), and there is no objection to clinical investigation from the UK Medicines and Healthcare products Regulatory Authority (MHRA) (Ref: CI/2022/0016/GB).

The study team has extensive experience of GAE, having completed the GENESIS study [6]. Patients will be recruited from orthopaedic surgery, rheumatology, general practice, physiotherapy, or self-referral. Participants must fulfil all inclusion criteria and none of the exclusion criteria (Table 1). Provided that they meet the eligibility criteria, patients with prior GAE may participate in the trial for the opposite knee. Participant consent form is provided in Online Appendix A.

**Table 1:** Inclusion/exclusion criteria

Inclusion criteria	Exclusion criteria
Participant is willing and able to give informed consent for participation in the study	Rheumatoid arthritis
Age ≥ 45 years	Infectious arthritis
Grade 1–3 knee OA on X-ray as per Kellgren–Lawrence Grading Scale	Severe (Grade 4) knee OA on Kellgren–Lawrence scale
Knee pain for ≥ 3 months that is resistant to conservative non-surgical treatment (e.g. physiotherapy, steroid injections, weight-loss programs, platelet-rich plasma injections)	Renal impairment (eGFR < 45, from medical records or blood test)
Able to lie flat for ≥ 6 h, assessed by asking how participants sleep (bed, chair recumbent, semi-recumbent) and what prevents them from lying flat overnight (breathlessness, back pain, etc.)	Bleeding diathesis (INR > 1.6, platelets < 50,000) or other bleeding risk, such as warfarin that cannot be stopped easily (e.g. patients with metallic heart valves) assessed by asking the patient and from medical records
Baseline VAS (visual analogue scale, 0–100) ≥ 50	Requires oxygen on ambulation
	Life expectancy < 1 year
	Communication difficulty due to language barriers
	Contraindication to magnetic resonance imaging (MRI)
	Known allergies to barium sulphate, 3-aminopropyltrialkoxysilane, polyphosphazene or IV radiopaque contrast agent
	History of peripheral arterial disease with intermittent claudication and/or rest pain
	Pregnancy or positive pregnancy test (due to fluoroscopy radiation exposure)
	Any other significant disease or disorder which, in the opinion of the recruiting physician, may put the participants at risk because of participation in the study, or may influence the result of the study or the participant’s ability to participate

### Interventions

#### GAE/Sham Procedure

Participants will be randomly allocated (1:1) to GAE or sham treatment using a computational generator created using code in Excel. The generator is maintained on a password protected research server. On the procedure day, an unblinded trained radiographer will inform the staff performing the study procedure of the participant’s allocation. At the 1-month and 6-month visits, participants will be asked which group they believed they were allocated to in order to assess accuracy of blinding. At 6 months post-randomisation, the participants and the study team will be unblinded. 6 months was chosen for unblinding as we know from prior studies that the median time for participants to reach maximum benefit from GAE is at least 3-months [10]. More importantly, we need a time period long enough to formally capture the placebo effect, whilst considering a time reasonable for participants to remain in the sham arm to maximise study recruitment, and minimise drop out.

GAE will be performed using 100-micron Embozene™ embolisation particles and following previously described methods for GAE [6]. The pathological embolisation target of the genicular arteries that supply the diseased portion of the knee is determined by patient symptoms and classical hyperaemic blush on angiography. All genicular arteries pertaining to the patients pain/synovitis demonstrated on preceeding contrast-enhanced MRI will be interrogated. Cone-beam CT (Allura FD20, Philips, Amsterdam) with contrast (6 ml of 100% contrast, 0.3 ml/s, 6 s delay) will be performed to confirm the hyperaemic target. A sports ice pack will be placed on the skin surface of the knee corresponding to the area to be embolised, minimising non-target embolisation to cutaneous branches by temporary vasoconstriction. The vessels will be embolised with 0.1–0.3 ml of embolic agent at a time to prune the abnormal vessels. The sham procedure will be identical to the GAE procedure, except that 2 ml heparinised 0.9% saline, instead of the embolic agent, will be slowly injected. Participants will be monitored for 4 h post-procedure.

Follow-up data will be collected at 1, 3, 6, 12, and 24 months post-randomisation (Table 2).

**Table 2:** Participant timeline

Timepoint	Study period														
	Enrolment	Allocation and treatment		Post-allocation						Post-treatment crossover to GAE after unblinding					
		Pre-Tx	Tx	1 week	1 mos^a^	3 mos^a^	6 mos^b^	12 mos^b^	24 mos^b^	1 week	1 mos^a^	3 mos^a^	6 mos^b^	12 mos^b^	18 mos^b^
*Enrolment*															
Informed Consent	X														
Demographics, medical history	X														
*Interventions*															
Randomisation		X													
GAE			X												
Sham			X												
Crossover to GAE							X								
*Assessments*															
Eligibility confirmation	X														
KOOS, VAS	X				X	X	X	X	X		X	X	X	X	X
Analgesia questionnaire	X				X	X	X	X	X		X	X	X	X	X
EQ-5D-3L	X						X	X	X				X	X	X
Knee MRI		X					X	X					X	X	
Neuroscience assessments		X													
Pre-existing conditions		X													
PROM				X						X					
Opinion of intervention					X		X								
AE check					X	X	X	X	X	X	X	X	X	X	X
Study completion									X						X
Durability of GAE									X						X

^a^** ± **5 days

^b^** ± **10 days

At 6 months post-randomisation, after unblinding, sham participants will have the option to undergo the GAE procedure (crossover). Participants electing to cross over will have 18 months of post-GAE follow-up.

Subjects may use analgesia, as indicated, throughout the study. Analgesia use will be collected from a participant self-reported analgesia questionnaire.

#### Pain, Function, and Quality of Life Assessment

Knee Injury and Osteoarthritis Outcome Score (KOOS) is a validated questionnaire used within the orthopaedic literature to assess the effect of interventions on the perceived severity of knee OA [12] and has previously been reported in the GAE literature [5, 6]. KOOS is intended to be used over short- and long-term time intervals, to assess changes from week to week induced by treatment (medication, operation, physical therapy) or over years following a primary injury or OA. KOOS is responsive to change following non-surgical and surgical interventions (Online Appendix A). The primary outcome measure for the current study is improvement in the 4 Knee Injury and OA Outcome Score subscales (KOOS_4_), covering pain, symptoms, activities of daily living, and quality of life at 6 months in patients with mild to moderate knee OA. Sports and recreation were the most inconsistently reported measure from Genesis, hence its exclusion [6]. Total KOOS will be analysed as a secondary endpoint. The Minimum Clinically Important Difference (MCID) will be used to define clinical success. A 10-point increase in KOOS scores will be used to define the MCID based on the work by Roos [6, 12]. The proportion of participants achieving the MCID at 6 months will be compared between the GAE and sham treatment for all 5 KOOS subscales. In addition to the quality of life assessment from the KOOS questionnaire, the EuroQoL-5D (EQ-5D-3L) will be completed.

A visual analogue scale (0–100) will also be used to assess pain in the affected knee. This will be performed at baseline, with participants with a VAS < 50 excluded.

Analgesia use will not be controlled throughout the study. Participants will be asked if they are taking analgesia specific to their knee pain at follow-up, enabling longitudinal trends in analgesia use to be compared to treatment response following GAE or the sham procedures.

Pain, function, analgesia, and quality of life assessment data will be prospectively collected using a purposely designed Electronic Data Capture System (EDC).

#### Psychosocial Pain Assessment

Prior to the embolisation procedure, participants will attend a neuropsychological presurgical assessment. Previous research has demonstrated a role for central sensitisation in the maintenance and chronification of Osteoarthritis [11]. It is hypothesised that characterisation of the likely contribution of such processes on an individualised basis will help to better account for treatment response. Participants will undergo brain functional magnetic resonance imaging (fMRI), quantitative sensory testing, a short behavioural experiment and fill out questionnaires assessing personality and emotional reactivity. The study will examine measures previously shown to predict the development of chronic pain, including neural (e.g. resting state connectivity of amygdala and ventral striatum and levels of metabolites, such as GABA or glutamate, within pain modulatory regions) and behavioural (conditioned pain modulation, temporal summation) biomarkers [13]. To examine psychological mechanisms, participants will complete a well-established behavioural experiment called the Wisconsin Card Sorting Task (WCST). The WCST is a useful behavioural quantifier of psychological flexibility and may be a useful variable of interest when attempting to stratify patients that achieve optimal clinical outcomes from this surgical procedure. To measure personality and emotional or pain reactivity, the following will also be administered: Pain Catastrophizing Scale, Committed Action Questionnaire, the Patient Health Questionnaire, Intolerance of Uncertainty measure, the Generalised Anxiety Disorder Inventory, the Five Factor Mindfulness Short-form Questionnaire, Psychological Inflexibility in Pain Scale, Pain Interference measure, and the Pittsburgh Sleep Quality Index (Online Appendix As). These will be used to model individual differences in treatment response (as measured using the KOOS and VASs).

#### Knee MRI

Participants will have *T*2-weighted, *T*1-weighted, STIR (Short Tau Inversion Recovery), and contrast-enhanced MRI sequences of the affected knee. Whole-Organ Magnetic Resonance Imaging Score (WORMS) will be used to provide a semi-quantitative non-invasive assessment of synovial hypertrophy and neo-vascularisation of the treated knee at baseline, 6, and 12 months post-procedure [14]. Images will be assessed by two independent consultant radiologists with training in WORMS, who are blind to treatment arm. Inter-observer agreement will be calculated.

### Outcome Measures

The research staff collating follow-up data will be blind to treatment allocation (double blind).

### Primary Outcomes

1. *Efficacy* Change in mean score on 4 Knee Injury and OA Outcome Score subscales, covering pain, symptoms, activities of daily living, and quality of life (KOOS4) at 6 months post-randomisation.

### Secondary Outcomes with Hypothesis Testing

1. Clinical success: Proportion of subjects reporting ≥ 10-point improvement in KOOS scores, where a 10-point change from baseline is defined as the minimum clinically important difference (MCID) at 6 months post-randomisation.
2. Pain relief: VAS change through 6 months post-randomisation.

### Secondary Outcomes with Descriptive Statistics

1. *OA symptom relief* Proportion of subjects reporting ≥ 10-point improvement in KOOS scores for each of the 5 KOOS subscales at 6 months post-randomisation
2. *Safety* The proportion of subjects experiencing relevant adverse events that are possibly, probably, or definitely related to treatment received, GAE or Sham, from randomization through 6 months post randomization will be summarized by severity and compared between GAE and sham arms [15].
3. *Synovial hypertrophy and neo-vascularisation* Whole-Organ Magnetic Resonance Imaging Score (WORMS) [14] at 6 months post-randomisation
4. *Neural and behavioural indicators of predisposition to central facilitation of pain* Three assessments quantifying (1) sensory pain profiles, (2) psychological profiles, (3) individual differences in neurochemistry and functional connectivity, with psychological profile quantified and calibrated for pain sensitivity and modulatory ability at 6 months post-randomisation
5. *Quality of life* Pre- and post-intervention EuroQoL-5D (EQ-5D-3L) for GAE versus non-crossover sham participants at 6 months post-randomisation
6. *Patient-reported acceptability of study procedure* Questionnaire 1 week after GAE

### Exploratory Outcomes

1. *Analgesia intake reduction from baseline* Analgesia questionnaire through 6 months post-randomisation
2. *Durability* Time from GAE procedure to next invasive intervention for knee OA
3. *Comparative cost-effectiveness* Payer-perspective health economic analysis of GAE versus standard of care, including integration of EQ-5D-3L-based quality-adjusted life-years, and durability.
4. *GAE group outcomes* KOOS, EQ-5D-3L, analgesia questionnaire, and VAS at 1, 3, 6, 12, and 24 months post-randomisation
5. *PROMs for sham treatment without crossover versus GAE* 12 and 24 months post-randomisation
6. *Imaging changes GAE versus sham* WORMS at 12 and 24 months post-randomisation
7. *Sham group outcomes* KOOS, VAS, and analgesia questionnaire diary at 1, 3, 6, 12 (crossover), and 18 months (crossover); EQ-5D-3L at 6, 12 (crossover), and 18 months (crossover); MRI at 6 and 12 months (crossover) post-randomisation.

### Sample Size

Alongside a sham surgery placebo arm, a high-quality systematic review of surgical RCTs was used as a suitable benchmark to inform the sample size and power calculation [16, 17]. With a 0–100 scale for the primary endpoint of KOOS_4_, assuming a 1:1 randomisation, a mean difference of 6.4 (i.e. effect size) between GAE and sham treatments, a common standard deviation of 10 (a Cohen’s D of 0.64), an alpha error of 2-sided 0.05, and a power of 0.85, the 2-arm total sample size requirement is 88 subjects (44 each arm) by Normal approximation. Allowing for a participant dropout rate of 20%, the enrolment goal is 110 participants, approximately 55 in each study arm. Study activities (Table 2) will take place at a single academic centre in the UK.

### Safety

The investigator will oversee study safety, including documenting and assessing all of participants’ AEs, regardless of severity or attribution. The number and type of serious AEs (SAEs) will be evaluated regularly, and procedure-related SAEs will also trigger assessment of whether any changes are needed to the protocol, patient informed consent, or other study documents or procedures.

After 5 subjects underwent GAE, GAE, and sham procedures stopped. A short safety and performance report was submitted to the MHRA for review. The MHRA made the determination that the study could resume based on this initial analysis.

### Statistics

#### Endpoints

All efficacy analyses will be performed on the intention-to-treat population (all randomised subjects).

Change of KOOS4 (mean of pain, symptoms, activities of daily living, and quality of life subscores) will be compared between GAE and sham treatment arms in an ANCOVA model (vs. treatment arm indicator and baseline KOOS4 score). A secondary ANCOVA model will further include pain medication taken through 6 months.

The proportion of subjects achieving the MCID (10 points) will be compared between the sham treatment and GAE arms by Fisher’s exact test for all 5 KOOS subscales.

Subjects’ 6-month VAS will be compared between GAE and sham treatment arms in an ANCOVA model (vs. treatment arm and baseline pain score). Should the VAS be generally low, score will be dichotomised to with (> 0) or without pain (= 0), and a logistic regression analysis will be performed.

All 5 subscales of the KOOS questionnaire will be analysed at 6 months follow-up. Each sub-scale will be compared between the GAE and sham arms in an ANCOVA model (vs. treatment arm indicator and the baseline sub-scale score). An additional analysis will further include investigational site in the model.

All remaining endpoints will be descriptive, and formal hypothesis testing will not be performed. Continuous data will be reported as mean, standard deviations, median, minimum, and maximum. Categorical data will be summarised as frequency and proportions.

The proportions of subjects experiencing relevant adverse events that are possibly, probably, or definitely related to treatment received, GAE or sham treatment, from randomisation through 6 months post-randomisation will be summarised by severity and compared between GAE and sham arms.

## Discussion

This study will be the largest double-blind RCT to evaluate a permanent-embolic for GAE in mild to moderate knee OA. The embolisation of abnormal blood vessel formation is hypothesised to reduce the knee OA symptoms that impact OA patients’ quality of life. The trial design will make it possible to assess efficacy of GAE whilst distinguishing the placebo effect from the postulated treatment effect. The results of this study will improve the generalisability of GAE data and provide high-level evidence on the safety, efficacy, and economic feasibility of the technique. GENESIS 2 will also provide imaging data from contrast-enhanced MRI. Direct comparison between GAE and a sham procedure will be possible, enabling assessment of imaging correlates for clinical success or failure, which may help optimise patient selection.

Pain is an extremely complex entity to measure and alter. To explore the multifactorial elements of pain perception relevant to knee OA, this trial builds on prior work [6] investigating the use of predictive pre-surgical neuropsychological assessments to identify markers for vulnerability to poor clinical outcome after GAE. This component of the trial may improve patient selection for GAE in the future.

Finally, this study will ascertain how participants perceive GAE as a procedure using a specifically designed patient questionnaire (PROM). This will provide invaluable data on how interventional radiologists can optimise procedural technique in order to maximise patient comfort and satisfaction.

## Supplementary Information

Below is the link to the electronic supplementary material.

Supplementary file1 (DOCX 8403 KB)

## Abbreviations

AE: Adverse event
GAE: Genicular artery embolisation
INR: International normalised ratio for prothrombin time
KOOS: Knee injury and osteoarthritis outcome score
MHRA: Medicines and healthcare products regulatory authority
MRI: Magnetic resonance imaging
OA: Osteoarthritis
SAE: Serious adverse event
VAS: Visual analogue scale (0–100)
WORMS: Whole-organ magnetic resonance imaging score
