# Supplementary material for: Genicular Artery Embolisation in Patients with Osteoarthritis of the Knee (GENESIS 2): Protocol for a Double-Blind Randomised Sham-Controlled Trial
Source: Cardiovasc Intervent Radiol. 2023 Jun 19;46(9):1276–82. doi: 10.1007/s00270-023-03477-z (PMC10471661; doi:10.1007/s00270-023-03477-z)

Appendix A

Consent Form


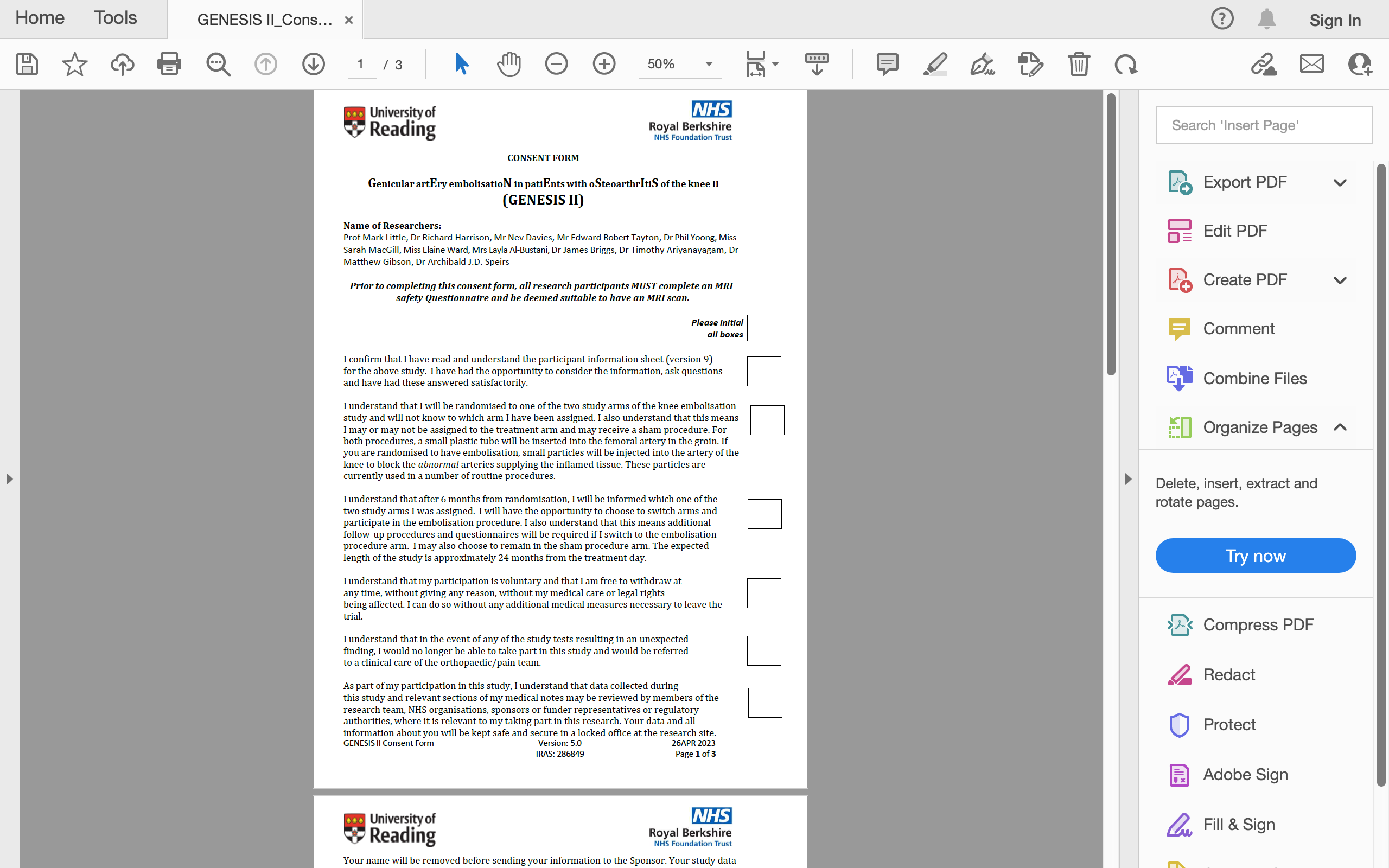


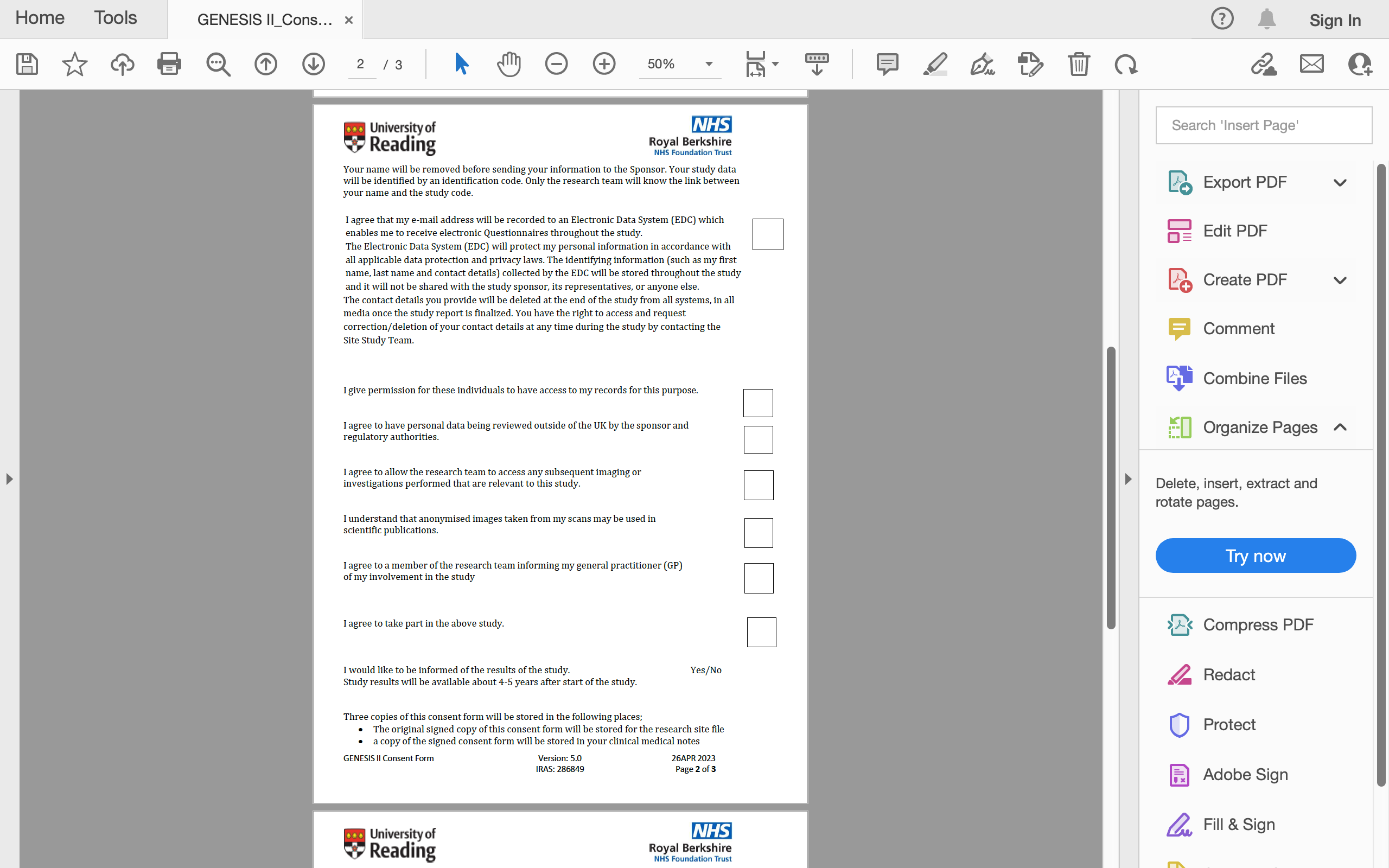


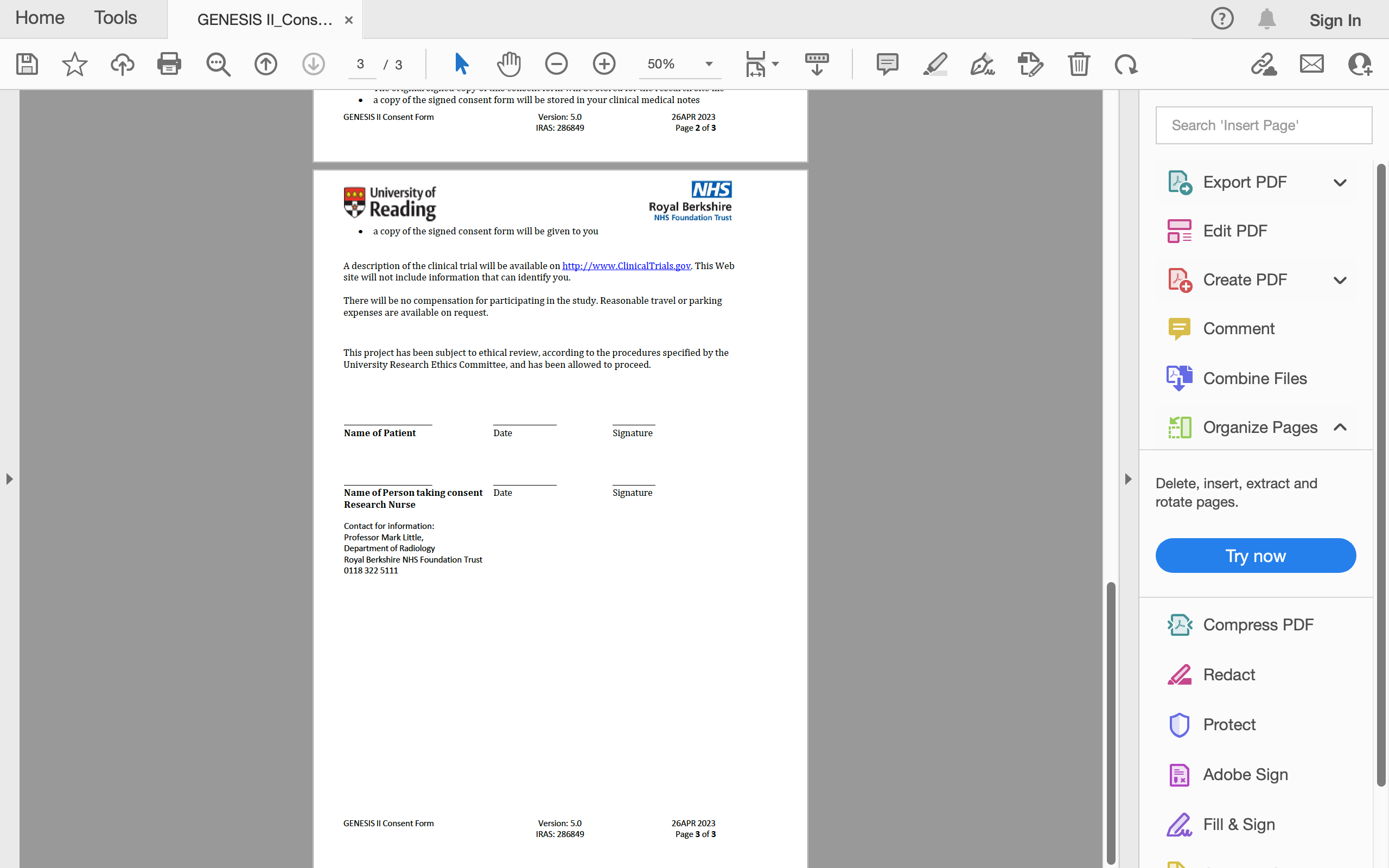


KOOS Questionnaire


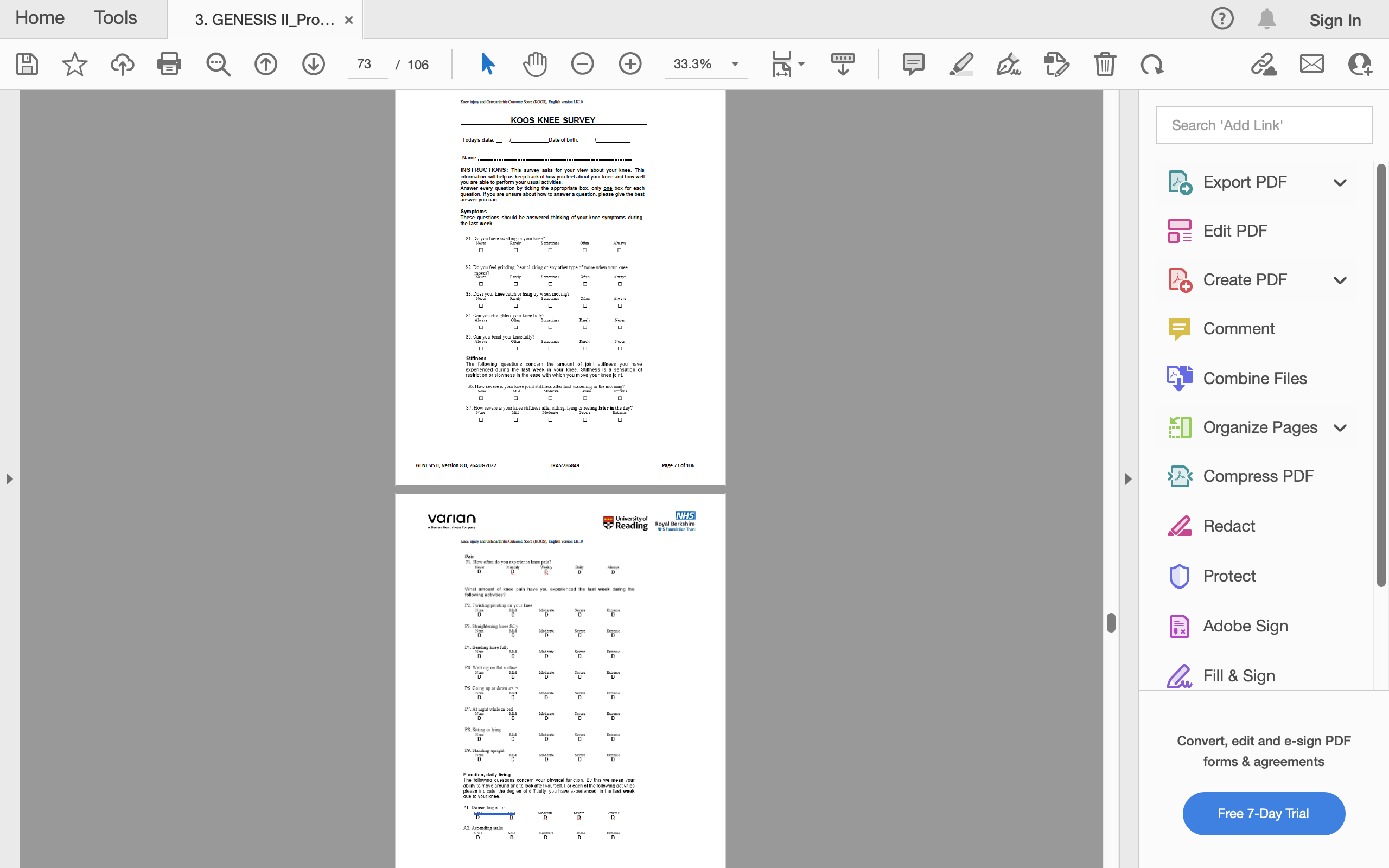


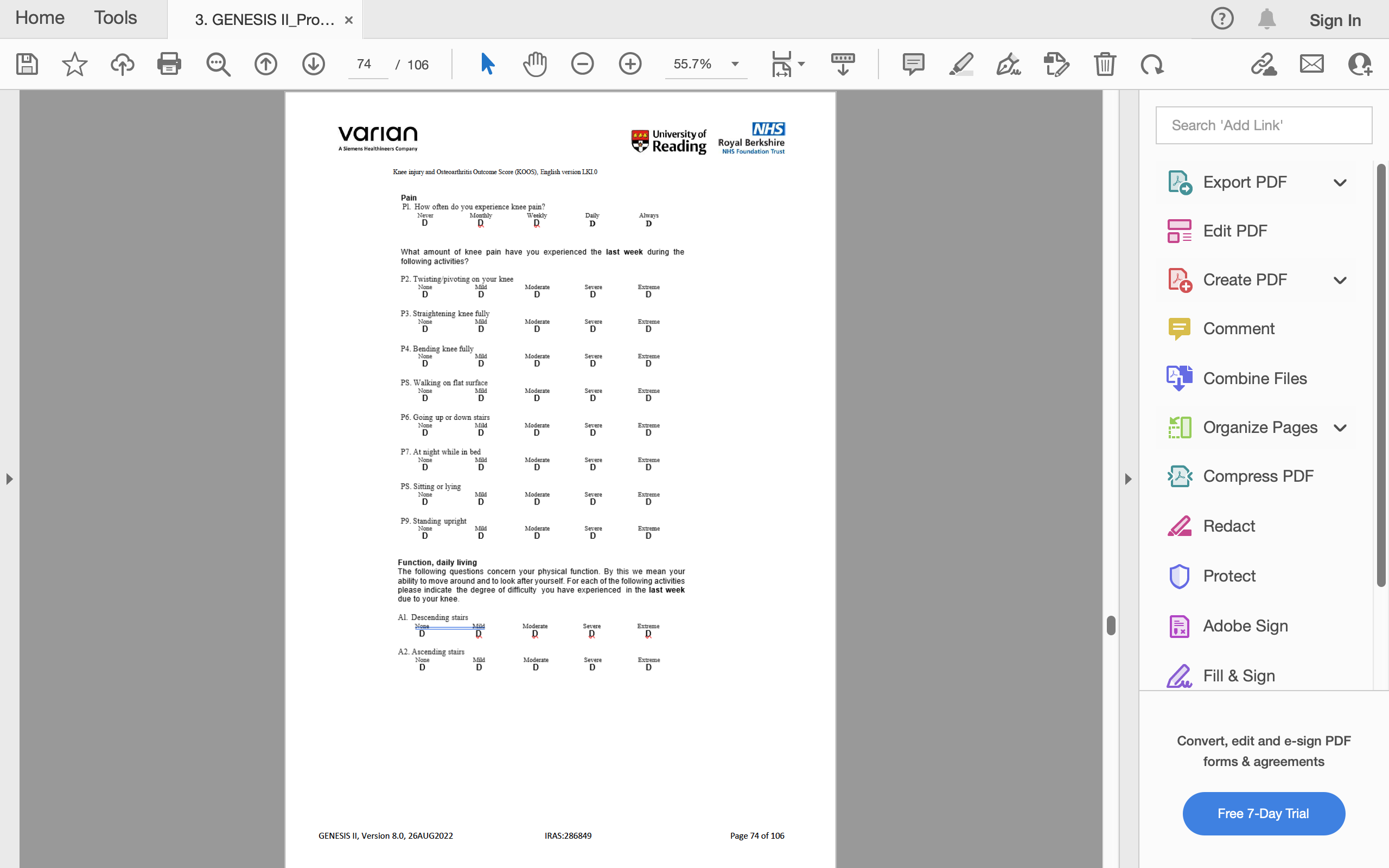


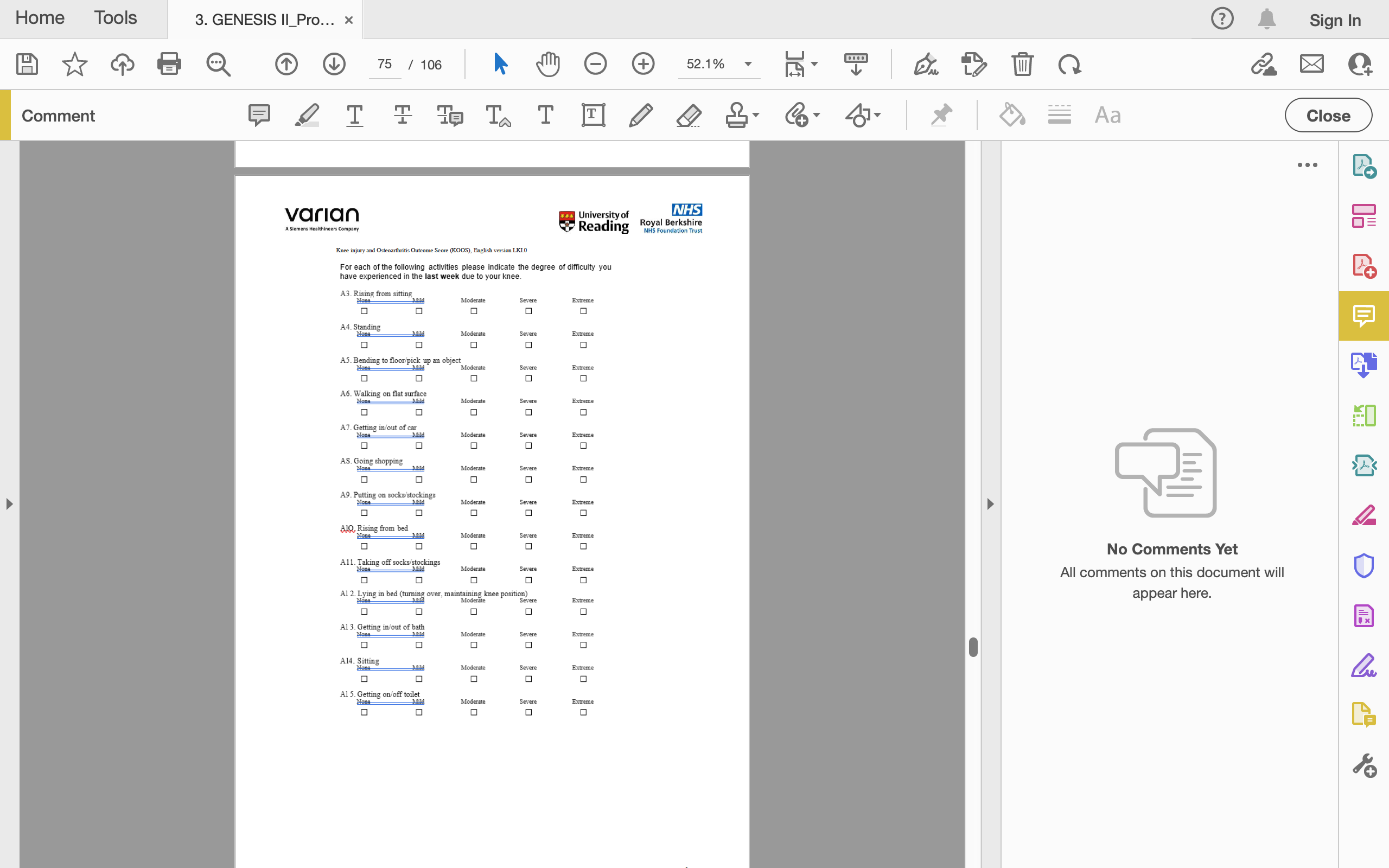


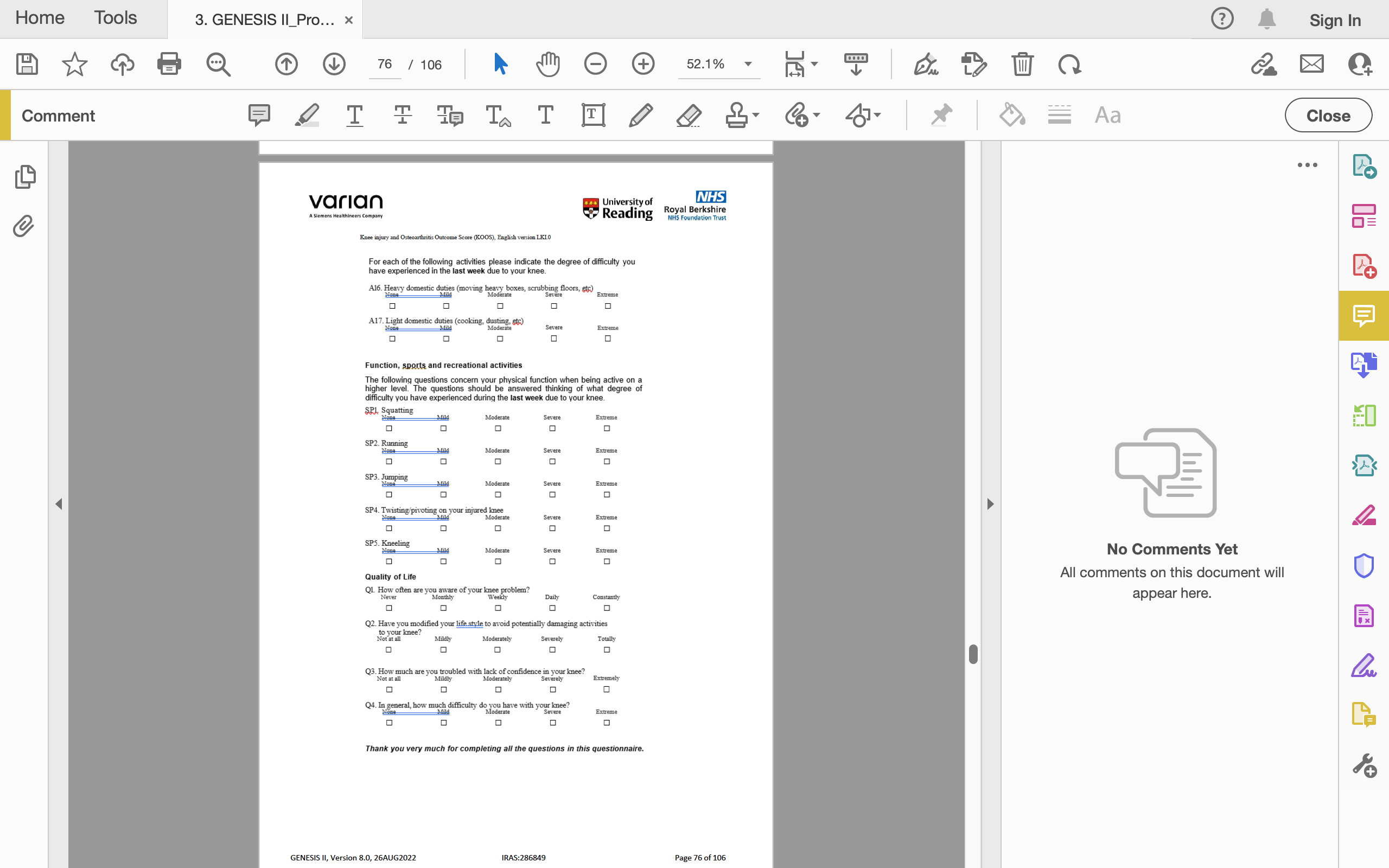


PSC Questionnaire


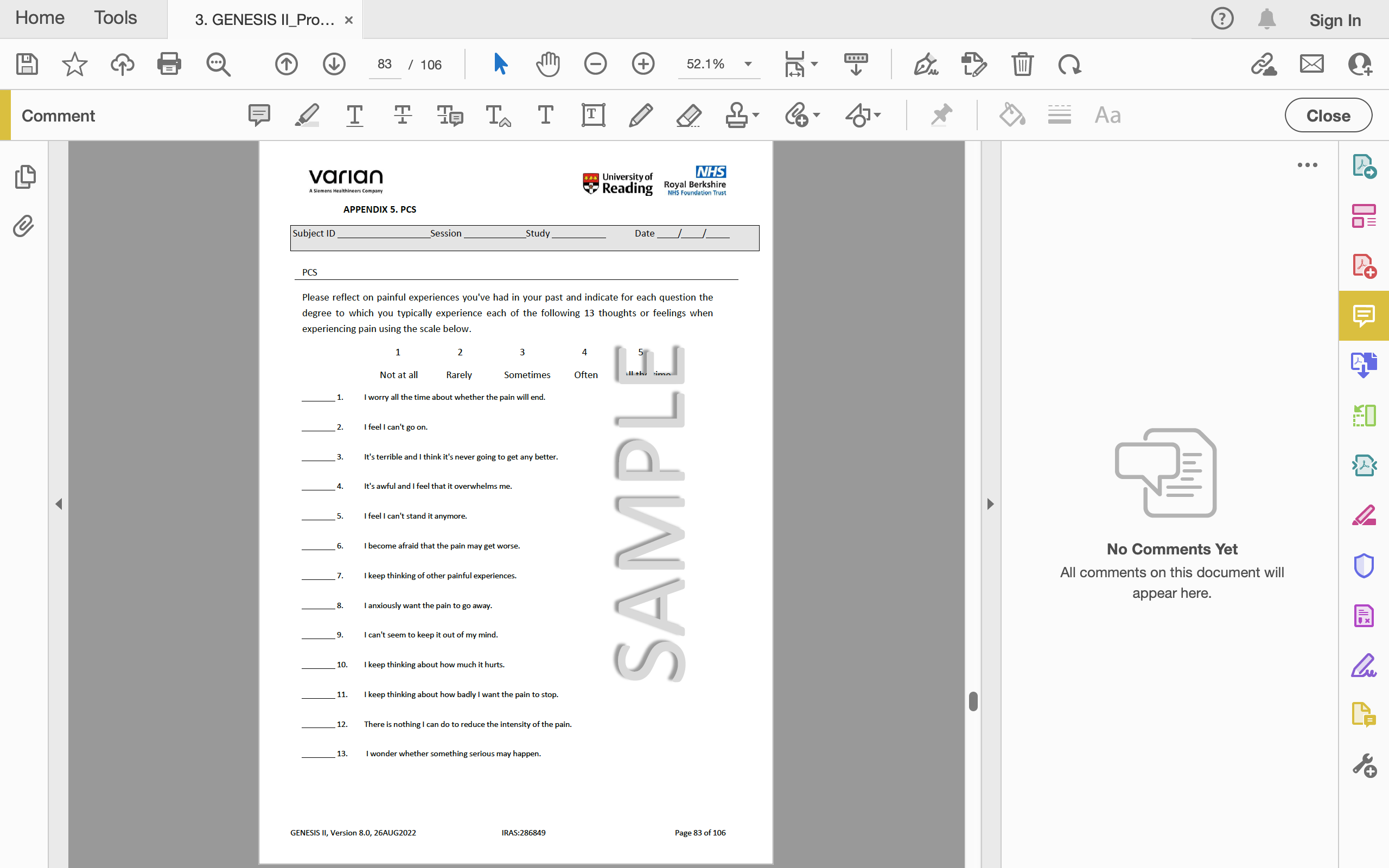


FFMQ-15 Questinnaire


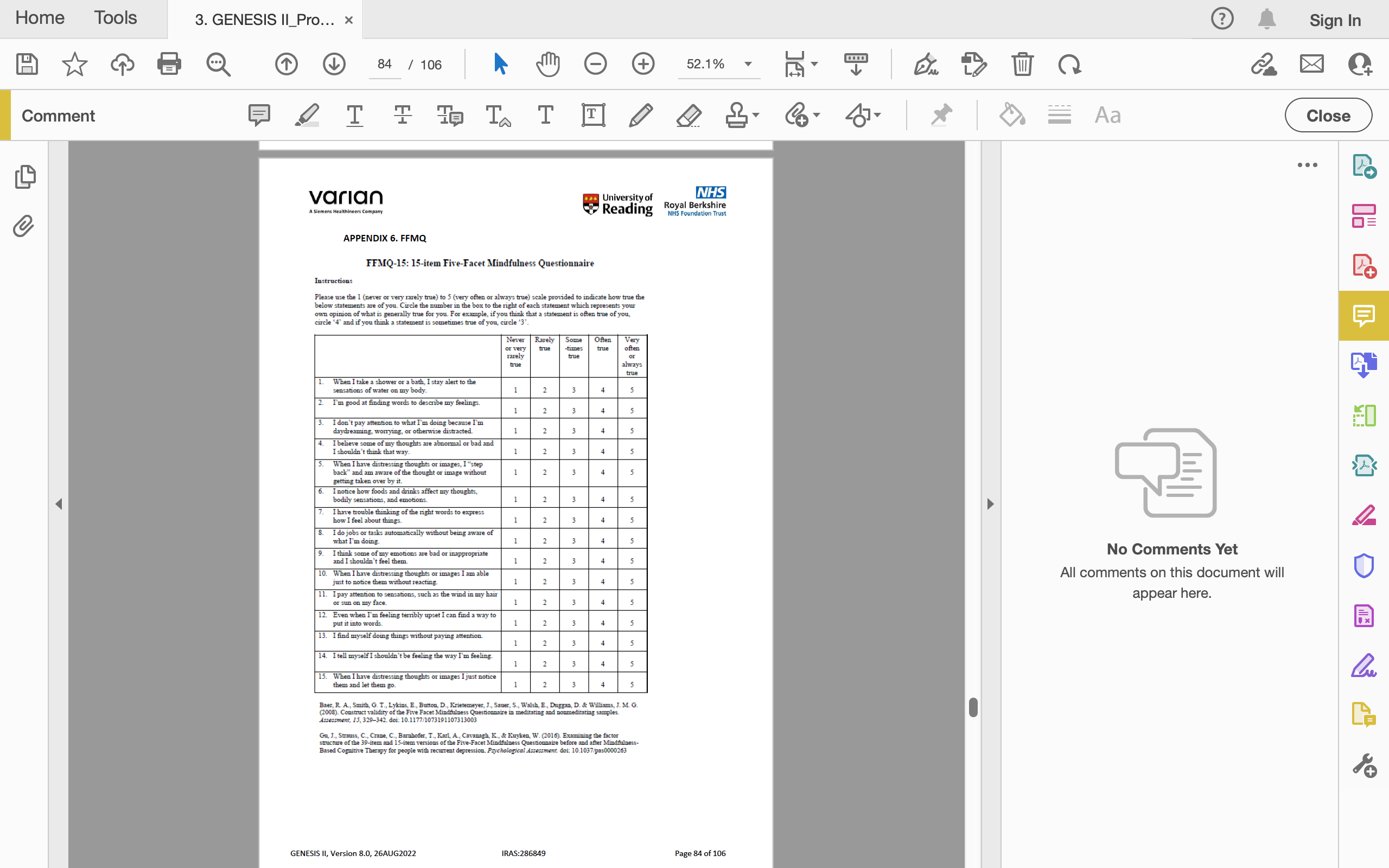


Patient Health Questionnaire


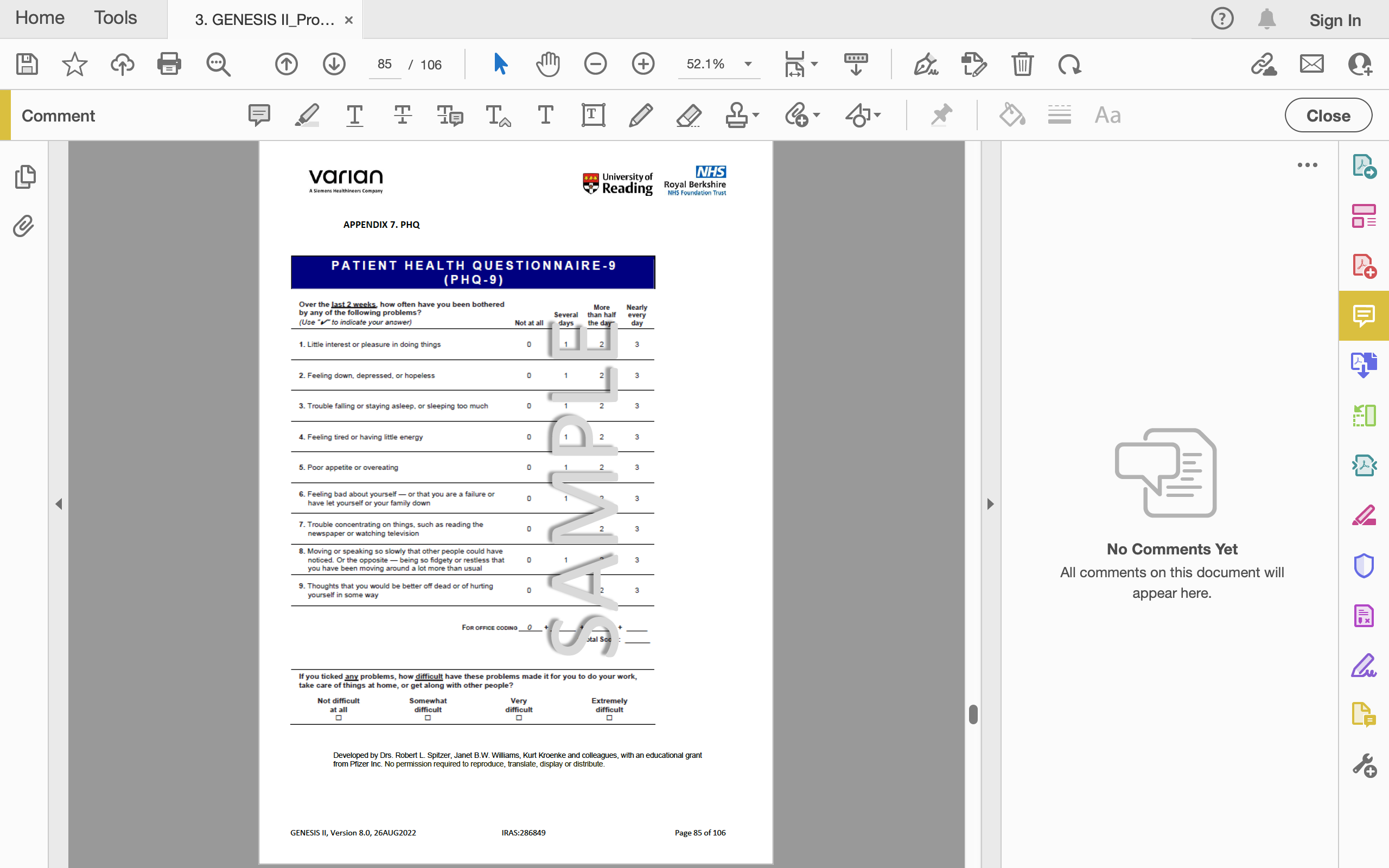


GAD Questionnaire


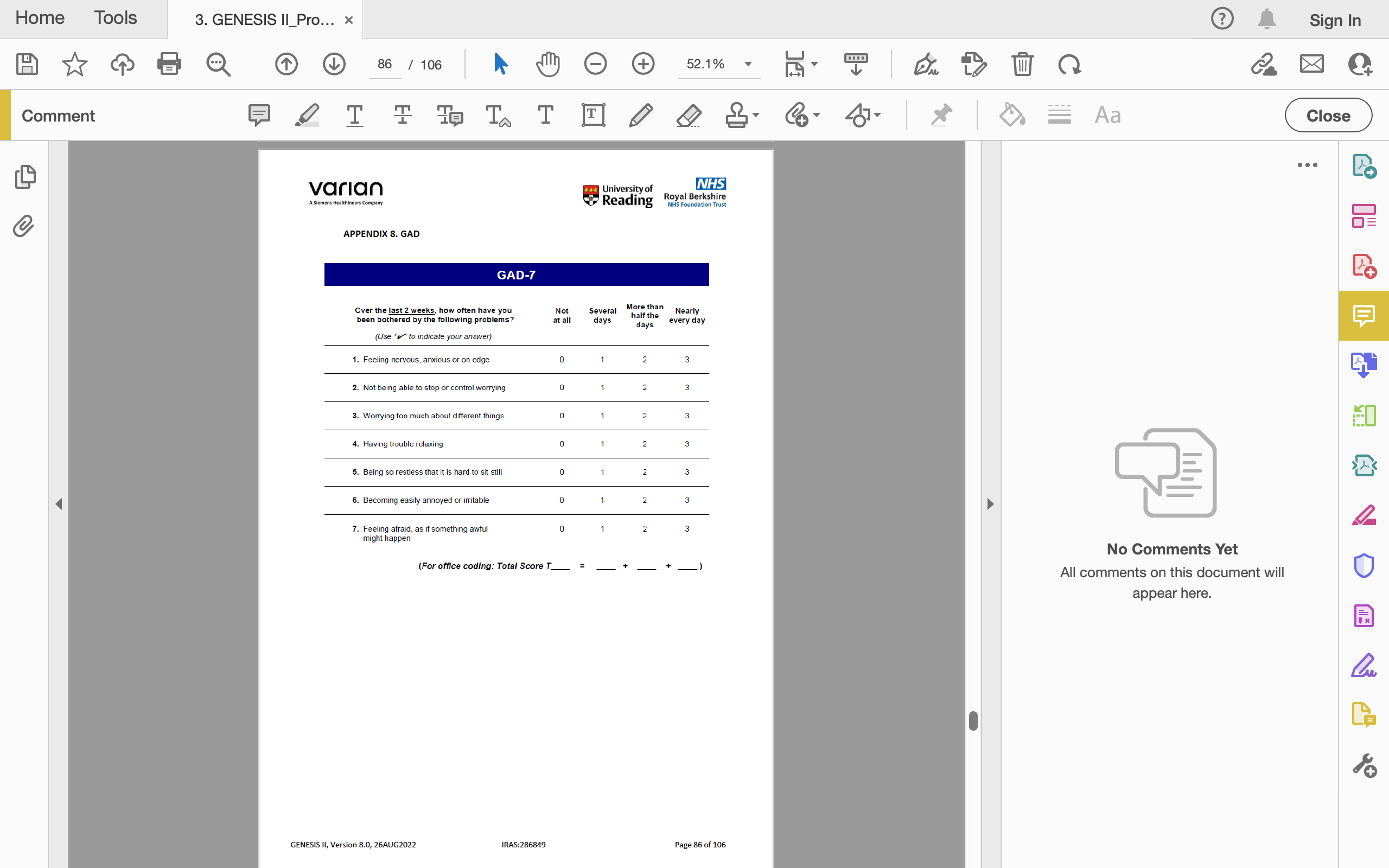


Committed Action Questionnaire


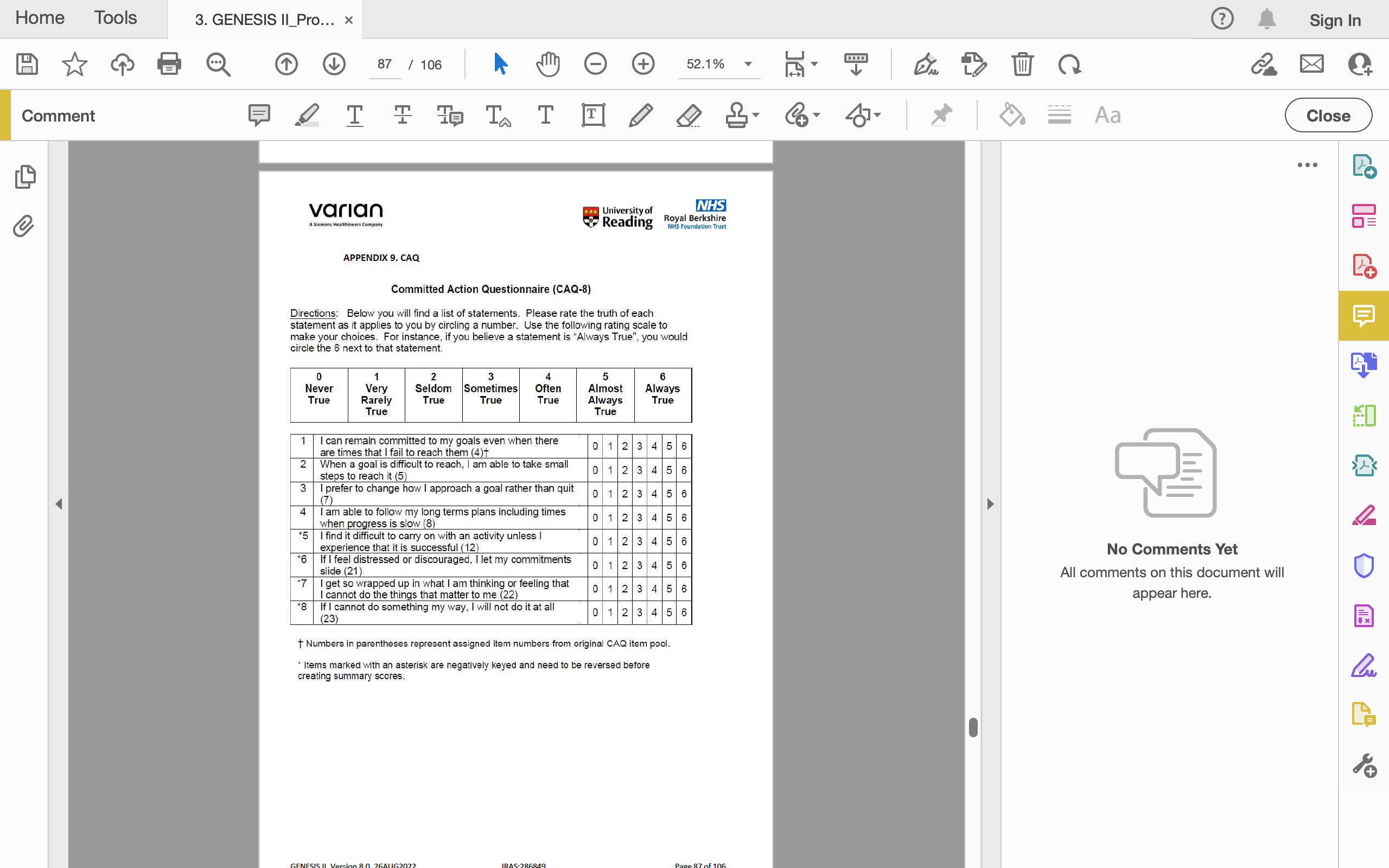


Pain Interference Questionnaire


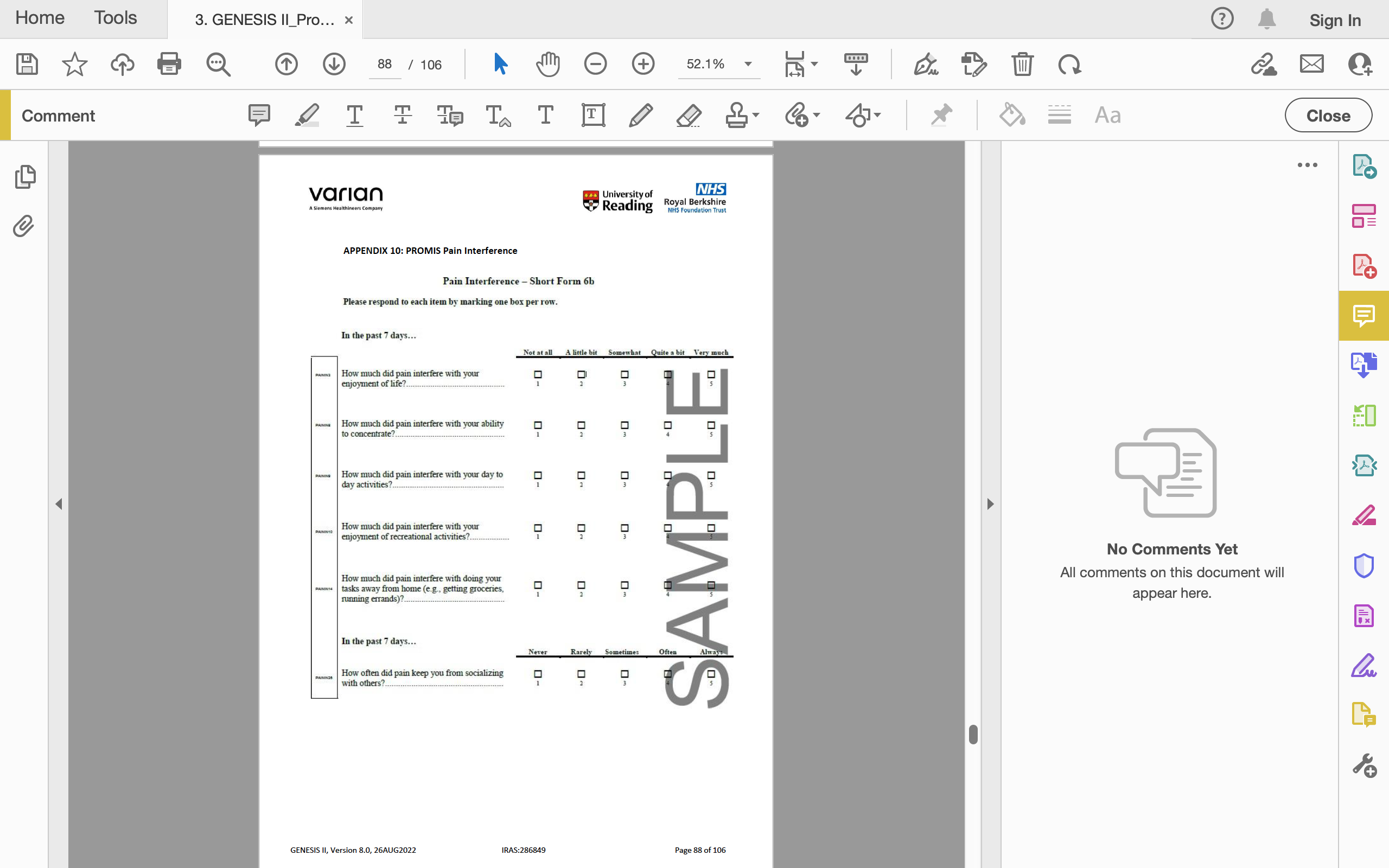


Intolerance of Uncertainty Questionnaire


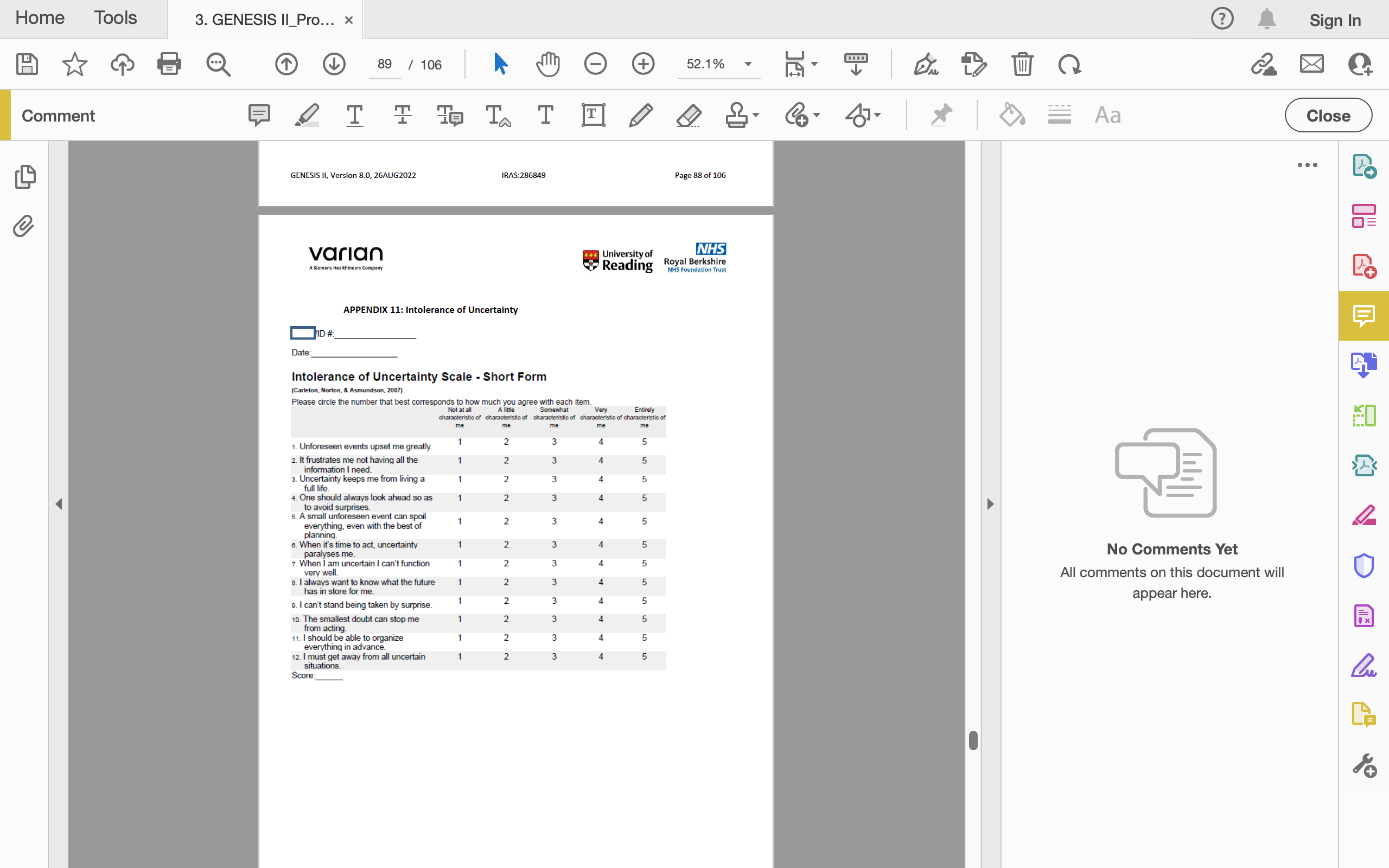


Pittsburgh Sleep Quality Index


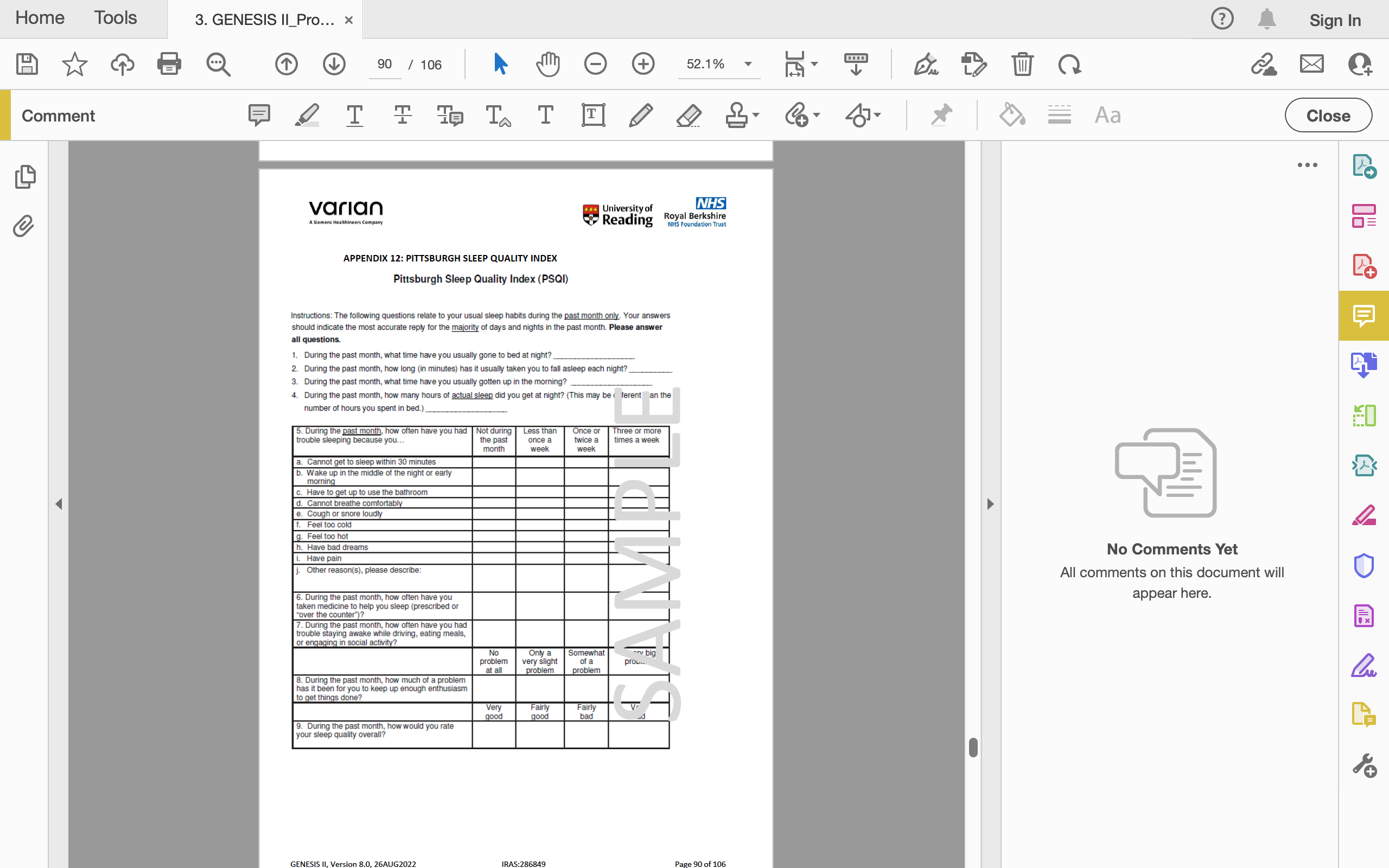


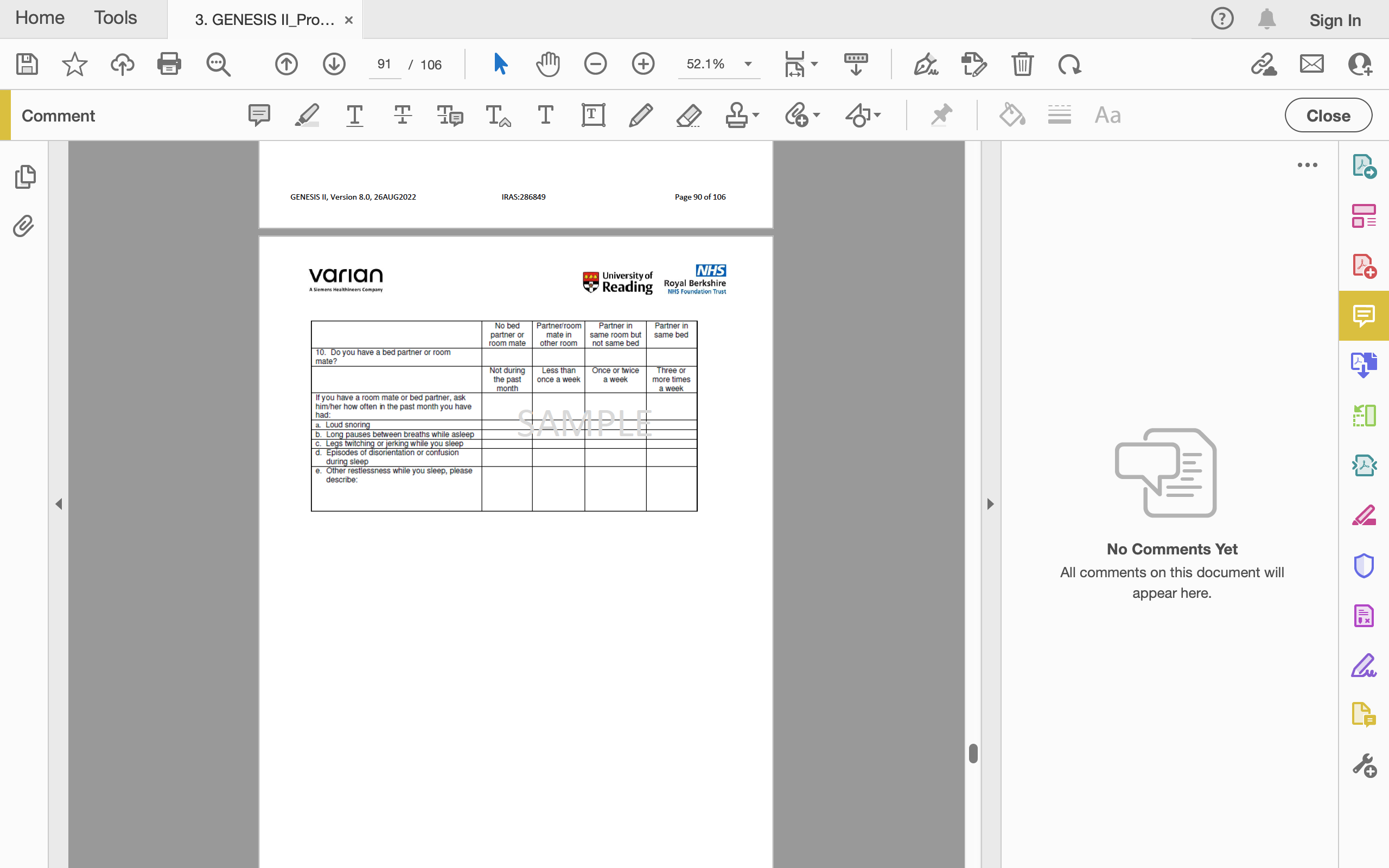


Pain Priors Questionnaire


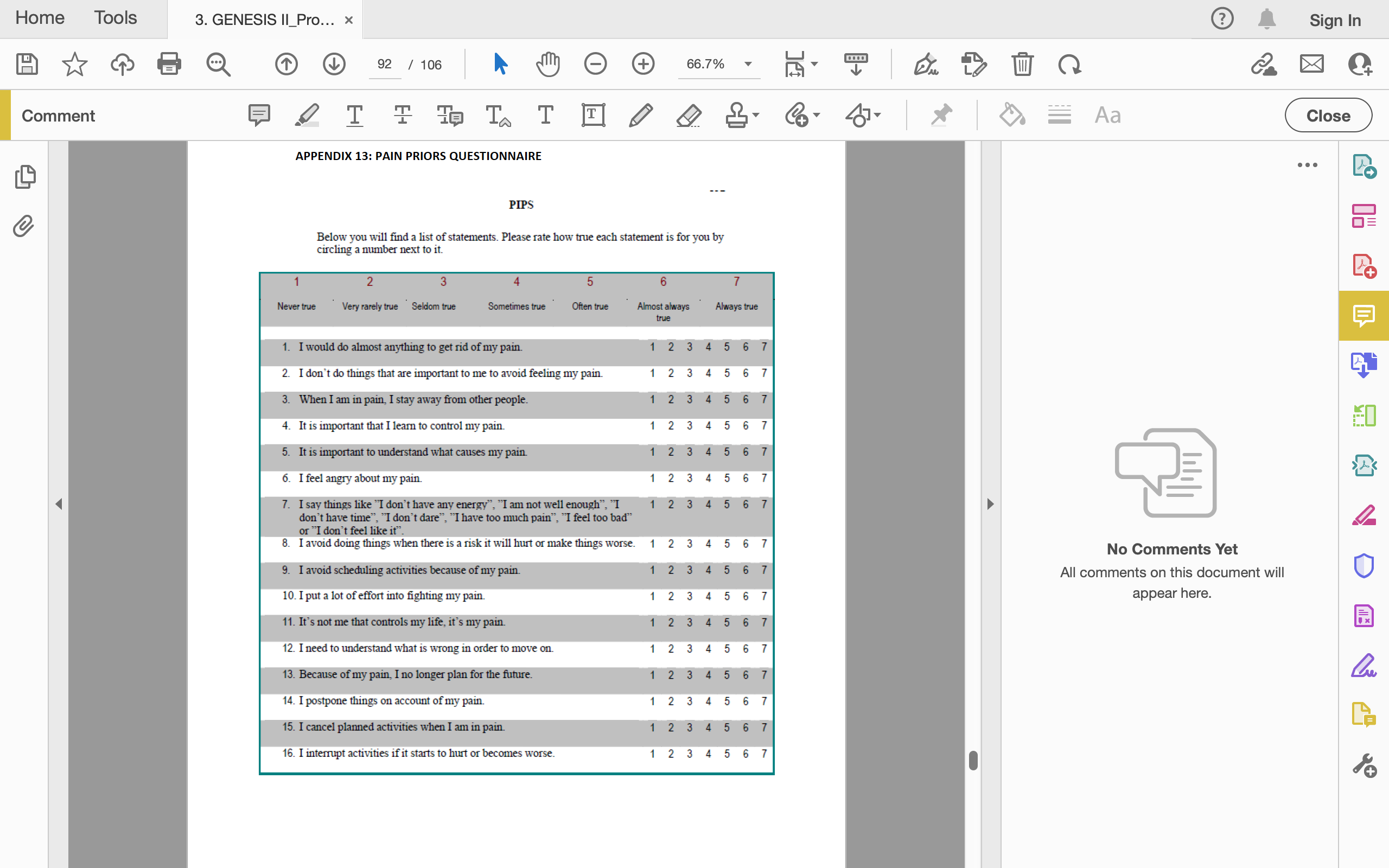

Supplement: Supplementary file 1 — Supplementary file1 (DOCX 8403 KB) [file 270_2023_3477_MOESM1_ESM.docx]
